# Changes in SeMSC, Glucosinolates and Sulforaphane Levels, and in Proteome Profile in Broccoli (*Brassica oleracea* var. *Italica*) Fertilized with Sodium Selenate

**DOI:** 10.3390/molecules18055221

**Published:** 2013-05-07

**Authors:** Ignacio Sepúlveda, Herna Barrientos, Andrea Mahn, Alejandra Moenne

**Affiliations:** 1 Department of Chemical Engineering, Faculty of Engineering, University of Santiago of Chile, Santiago 9170019, Chile; E-Mails: ignaciodsg@gmail.com (I.S.); herna.barrientos@usach.cl (H.B.); 2 Department of Biology, Faculty of Chemistry and Biology, University of Santiago of Chile, Santiago 9170022, Chile; E-Mail: alejandra.moenne@usach.cl

**Keywords:** selenium, myrosinase, proteomics, broccoli

## Abstract

The aim of this work was to analyze the effect of sodium selenate fortification on the content of selenomethyl selenocysteine (SeMSC), total glucosinolates and sulforaphane, as well as the changes in protein profile of the inflorescences of broccoli (*Brassica oleracea* var. *Italica*). Two experimental groups were considered: plants treated with 100 μmol/L sodium selenate (final concentration in the pot) and control plants treated with water. Fortification began 2 weeks after transplantation and was repeated once a week during 10 weeks. Broccoli florets were harvested when they reached appropriate size. SeMSC content in broccoli florets increased significantly with sodium selenate fortification; but total glucosinolates and sulforaphane content as well as myrosinase activity were not affected. The protein profile of broccoli florets changed due to fortification with sodium selenate. Some proteins involved in general stress-responses were up-regulated, whereas down-regulated proteins were identified as proteins involved in protection against pathogens. This is the first attempt to evaluate the physiological effect of fortification with sodium selenate on broccoli at protein level. The results of this work will contribute to better understanding the metabolic processes related with selenium uptake and accumulation in broccoli.

## 1. Introduction

Selenium is an essential trace element for humans and mammals since it ensures activity of key metabolic enzymes such as glutathione peroxidases, thioredoxin reductases and tetraiodothyrosine 5'-deiodinases [1]. The deficiency of selenium in humans causes a reduction in fertility, in immune and cognitive functions, hyperthyroidism and an increased susceptibility to cancer [2]. Selenium uptake in humans is mainly ensured by consumption of vegetables which contain selenium in the form of seleno amino acids and methylseleno amino acids. Interestingly, selenium is a scarce element in soil and it is not essential for plant viability. However, selenite and selenate salts, which are taken from the soil through the absorption pathway of sulfate, have demonstrated to improve the antioxidant status of plants [3]. Selenium is stored in plants as selenocysteine and selenomethionine, which are incorporated into proteins by replacing the standard amino acids [4,5]. In selenium hyperaccumulator plants of the *Brassicacea* family, selenium can also be found as non-proteinogenic amino acids such as selenomethyl selenocysteine (SeMSC), γ-glutamyl SeMSC and selenocystathionine [5]. In particular, seleno amino acids and methylseleno amino acids have high anticarcinogenic activity in mammals, and the highest activity is displayed by SeMSeC [6,7]. 

In addition to the ability of accumulating selenium, *Brassicaceae* plants synthesize sulfur and nitrogen containing secondary metabolites named glucosinolates, which derive from amino acids and are linked to a glucose residue [8]. Glucosinolates and their hydrolysis products act as protecting agents against herbivores and fungi in plants [9]. Glucosinolates are hydrolyzed by the enzyme myrosinase, releasing a glucose residue and the aglycone that spontaneously rearrange into isothiocyanates, nitriles and thiocyanates [8]. In particular, glucoraphanin, the major aliphatic glucosinolate found in broccoli, is hydrolyzed by myrosinase to yield sulforaphane, an isothiocyanate that exhibits a high anticarcinogenic activity [10]. In addition, selenoglucosinolates have a higher anticarcinogenic activity than thioglucosinolates, and are accumulated in selenium-fortified *B. napus* and broccoli [11]. Although the natural concentration of SeMSC in *Brassicacea* is relatively low, it can be significantly increased by fortifying the soil or culture media with sodium selenite or selenate [12]. In this sense, several works have determined that selenium fortification competes with sulfate uptake leading to a decrease in sulfur-containing compounds [13]. However, recent works have shown that selenium fortification increases the content of sulfur-containing compounds such as cysteine, glutathione and total glucosinolates [14] and also sulfur uptake in *Arabidopsis thaliana* and broccoli [15].

On the other hand, several proteomic studies about the effect of nutrient-induced stress on *Brassicaceae* plants have been performed. In particular, *B. napus* exposed to phosphorus deficiency showed changes in the synthesis of some proteins related with gene transcription, carbon metabolism, energy transfer, stress-responses and defense against pathogens [16]. In addition, transgenic *B. napus* that over-expressed nicotin amine synthase gene exposed to sodium excess showed changes in the synthesis of some proteins involved in salt tolerance, energy metabolism and defense [17]. Moreover, *B. napus* subjected to boron deprivation showed changes in the synthesis of proteins involved in carbohydrate and energy metabolism, stress response, amino acid and nucleic acid metabolism, among others [18]. Until now, few proteomic studies have been performed in broccoli [19] and no studies about the selenium-enrichment or deprivation on protein profile are currently available. 

In this work, we analyzed the effect of fortification with sodium selenate on the content of SeMSC, total glucosinolates and sulforaphane, as well as the changes in proteome profile in the inflorescence of broccoli. The aim of this work was to contribute to understanding the metabolic processes related with selenium uptake and accumulation in broccoli.

## 2. Results and Discussion

### 2.1. Selenate Fertilization Increases SeMSC Level But does not Affect Significantly Glucosinolates and Sulforaphane Levels in Broccoli

**Figure 1:**
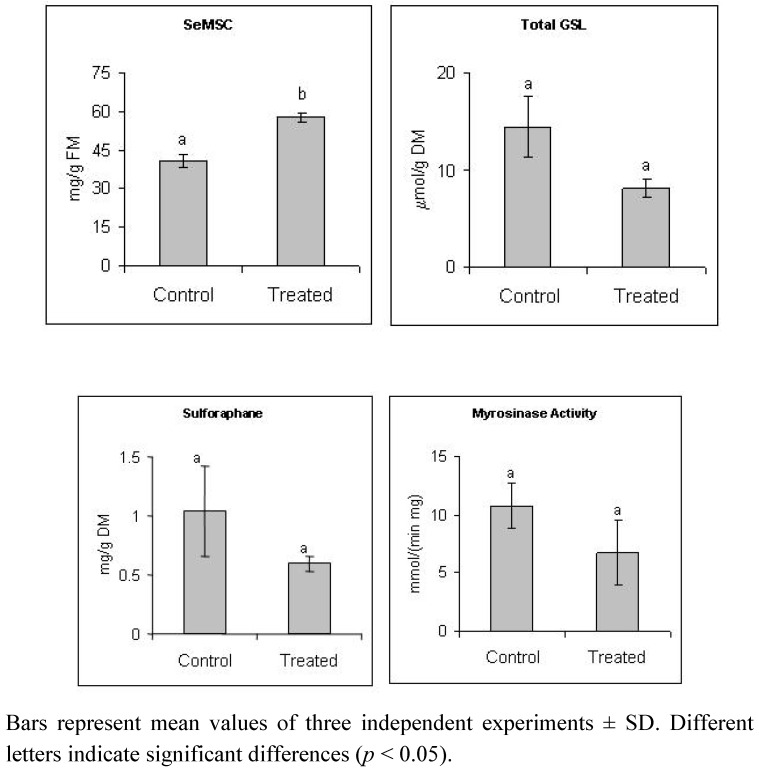
SeMSC, total glucosinolates and sulforaphane content and myrosinase activity in broccoli fertilized with water (control) or with 100 µM sodium selenate (treated).

Figure 1 shows the concentration of SeMSC, total glucosinolates, and sulforaphane in broccoli florets, as well as myrosinase activity. As expected, the SeMSC content was significantly higher in broccoli subjected to selenate fortification, showing a 143% higher concentration in the treated plants. This confirms that broccoli accumulates selenium taken from the soil as an inorganic salt in the form of seleno amino acid. Interestingly, glucosinolates and sulforaphane content were apparently not affected by selenate fortification, since no statistically significant differences (95% confidence level) were detected between control and treated plants, despite the competition between selenium and sulfur that occurs since both elements are incorporated through the same initial assimilation route [5]. This null effect of fertilization with selenium on sulfur uptake can be attributed to the comparatively low concentration of sodium selenate in comparison with the usual sulfur concentration in soil. These results agree with Hsu *et al.* [14], who demonstrated that broccoli can be fortified with selenium without reduction in total glucosinolates content, even when exceeding the level recommended for human consumption. Besides, myrosinase activity, which is the enzyme responsible for the hydrolysis of glucoraphanin (the main glucosinolate found in broccoli) to yield sulforaphane, showed no significant difference between control and treated plants. This agrees with the behavior observed for total glucosinolates and sulforaphane content. However, notwithstanding the statistical results, a clear trend towards decrease of sulforaphane and glucosinolates content is observed in Figure 1. The results from the statistical analysis may relate to the relatively low accuracy and precision of the analytical methods used for quantifying sulforaphane and glucosinolates, which are secondary methods and may be somewhat unspecific [14]. 

### 2.2. Selenate Fertilization Changes the Proteome Profile in Broccoli Florets

**Figure 2:**
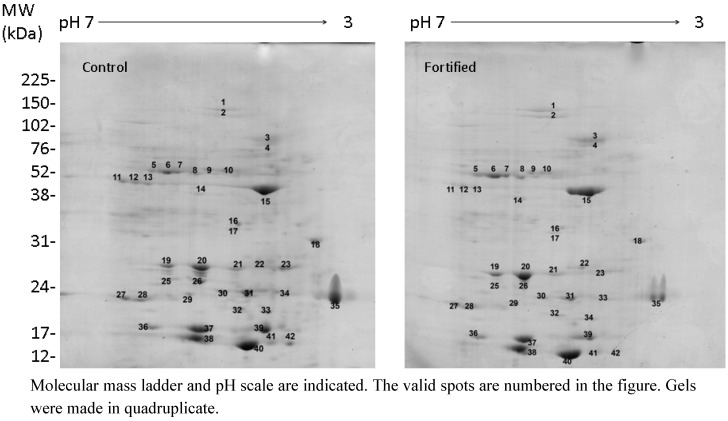
Representative 2D gel images of control and fortified broccoli.

The effect of fortification with sodium selenate on the protein profile of broccoli florets was investigated through two-dimensional gel electrophoresis (2D-PAGE) and mass spectrometry (MS). Figure 2 shows representative 2D-PAGE images of control and fortified broccoli. The valid spots (this is the spots that were present in all replicate gels of the same condition) are highlighted with numbers. The relative abundance of each valid spot was estimated as normalized volume, and the average normalized volume of each spot in each condition is presented in Table 1. This table shows that of the 42 valid spots detected, 16 decreased their normalized volume in selenium-enriched broccoli; 25 spots increased their normalized volume, and one was detected only in selenium-fortified broccoli. Statistically significant differences in the protein abundances were determined through a Student’s *t* test, al 95% confidence level. The results of the statistical analysis are given in Table 1. Here we observe that spots number 3, 10, 15, 20, and 28 significantly increased their relative abundance when broccoli crop was fortified with sodium selenate, in comparison with the control condition. The spots number 18, 23, 31, and 42 significantly decreased their abundance with selenium fortification. The protein spots whose abundance showed significant differences between fortified and control broccoli were identified by mass spectrometry. Table 2 shows the results of protein identification. Spots number 31 and 42 could not be identified likely because the broccoli proteome is not complete yet, and these proteins probably differ considerably from homologous proteins in Arabidopsis thaliana (the proteome data base where the search was performed). 

Up-regulated proteins were identified as proteins involved in general stress-responses such as a heat shock protein HSP70 (spot 3) and glutathione-S-transferase (spot 28), and in mitochondrial and chloroplast electron transport such as mitochondrial ATP synthase subunit β (spot 10), myrosinase-binding protein (spot 15), and chlorophyll-binding protein (spot 20) (Table 1). Down-regulated proteins were identified as proteins involved in protection against pathogens such as a β-1,3 glucanase (spot 18) and a photosystem II protein (spot 23). Down-regulated proteins 31 and 42 were not identified probably because the broccoli proteome is not complete yet or because they strongly differ from homologous proteins in *Arabidopsis thaliana*.

**Table 1:** Normalized spot volume (Means ± standard deviation) and statistical comparison (Student’s *t* Test) between fortified and control plants.

	Normalized Spot Volume		
Spot Nr.	Control	Fortified	*p*-value
**1**	6.3 ± 2.1	10.8 ± 5.4	0.172
**2**	5.5 ± 0.9	8.8 ± 2.9	0.073
3 *	8.9 ± 3.1	32.8 ± 6.3	0.001 *
**4**	6.1 ± 1.6	5.1 ± 1.6	0.449
**5**	9.0 ± 5.0	15.6 ± 2.3	0.055
**6**	34.3 ± 12.8	44.5 ± 9.8	0.252
**7**	13.9 ± 4.0	12.8 ± 4.8	0.727
**8**	8.2 ± 3.3	13.0 ± 2.5	0.059
**9**	10.5 ± 5.3	8.1 ± 2.1	0.437
10 *	10.0 ± 1.1	14.5 ± 1.1	0.001 *
**11**	12.0 ± 2.7	6.7 ± 3.6	0.077
**12**	10.4 ± 3.5	6.4 ± 2.2	0.105
**13**	9.7 ± 2.0	6.8 ± 1.8	0.079
**14**	3.6 ± 1.01	2.5 ± 1.2	0.296
15 *	104.1 ± 11.0	132.3 ± 8.8	0.007 *
**16**	5.8 ± 1.9	6.4 ± 1.7	0.633
**17**	4.9 ± 1.7	3.5 ± 0.8	0.206
18 *	21.2 ± 2.1	12.3 ± 1.2	0.000 *
**19**	21.8 ± 2.7	23.9 ± 2.3	0.332
20 *	57.0 ± 8.1	76.1 ± 4.8	0.015 *
**21**	18.5 ± 6.0	12.6 ± 2.7	0.123
**22**	9.8 ± 3.0	8.3 ± 1.7	0.403
23 *	25.6 ± 0.9	11.4 ± 5.2	0.002 *
**24**	5.9 ± 0.9	5.7 ± 0.6	0.397
**25**	7.9 ± 5.7	-	-
**26**	23.8 ± 11.2	1.8 ± 0.4	0.059
**27**	13.5 ± 3.5	16.1 ± 5.1	0.432
28 *	9.5 ± 6.5	19.5 ± 2.3	0.028 *
**29**	7.0 ± 1.7	5.6 ± 1.4	0.371
**30**	10.7 ± 2.4	9.1 ± 0.3	0.332
31 *	34.9 ± 2.7	26.9 ± 3.9	0.014 *
**32**	8.4 ± 3.7	5.2 ± 1.8	0.227
**33**	7.5 ± 4.6	5.1 ± 2.6	0.390
**34**	7.7 ± 4.4	3.9 ± 0.5	0.205
**35**	115.5 ± 17.1	93.8 ± 43.1	0.387
**36**	18.0 ± 1.1	19.2 ± 4.4	0.608
**37**	70.7 ± 7.6	60.8 ± 4.5	0.104
**38**	73.8 ± 8.3	84.4 ± 15.6	0.276
**39**	44.7 ± 8.6	38.3 ± 10.8	0.388
**40**	104.6 ± 22.7	125.8 ± 21.8	0.227
**41**	18.1 ± 7.6	15.0 ± 5.6	0.531
42 *	11.2 ± 2.0	6.6 ± 0.1	0.012 *

* Differentially expresses spots are highlighted with.

**Table 2:** Identification of protein spots that show significant differences in synthesis level between fortified and control plants.

SN	RC	Protein identification	Peptide sequences	Accession number	Theoretical		PS
					MW (KDa)	pI	
3	+	Heat shock protein 70KDa (*A. thaliana*)	FEELNMDLFR	gi|9294373	71.3	5	640
			NALENYAYNMR				
			TTPSYVAFTDSER				
			ARFEELNMDLFR				
			NAVVTVPAYFNDSQR				
			GVWEGQPHADIGRIDLGTTYSCVG				
			EYQEGIFESRTYSDNQPGVLIQV				
10	+	Mitochondrial F1 ATP synthase beta subunit (*A. thaliana*)	VVDLLAPYQR	gi|6686269	54.2	5.4	1060
			AHGGFSVFAGVGER				
			VGLTGLTVAEYFR				
			FTQANSEVSALLGR				
			LVLEVSHHLGQNVVR				
			DAEGQDVLLFIDNIFR				
			QISELGIYPAVDPLDSTSR				
			IQPESRAVGYQPTLASDLGAL				
			FHEPDTQREGLPPIMTSLEVQD				
			FHEPDTQREGLPPIMTSLEVQD				
15	+	Myrosinase- binding protein (*A. thaliana*)	VYVGQGDSGVVYVK	gi|9279646	32.3	4.8	97
18	−	β-1,3-glucanase (*O. europaea*)	AIETYIFAMFDENQK	gi|51507325	23.4	8.8	68
20	+	Chlorophyll protein (*B. oleracea*)	KEPFYGGIAYK	gi|27530934	22.2	6	322
			TTAQYLILPLSPR				
			LQPLCPLGISQSSVK				
			YGWGKEVDESSSASEEPAILV				
23	−	Photosystem II protein (*B. oleracea*)	FLVPSYR	gi|49359169	36.8	6.8	469
			EREDGIDYAAVTVQLPGGE				
			SDKTPDELGTGAEKVIGVFQSLQPS				
28	+	Glutathione S-transferase (*A. thaliana*)	VYGPHFASPK	gi|3201613	24.9	5.6	121
			YDLYALGVGDFPVIGSKLADLAHLPFT				

Sn = spot number; RC = relative change, protein identification is the most probable identity (and source) of the protein analyzed by mass spectrometry, peptide sequences are the sequences fat match with each peptide mass, accession number is the Genebank code that corresponds to the identified protein, theoretical MW and pI are the molecular weight and Isoelectric point of the protein estimated from the amino acid sequence, PS is the protein score in the Mascot database.

**Figure 3:**
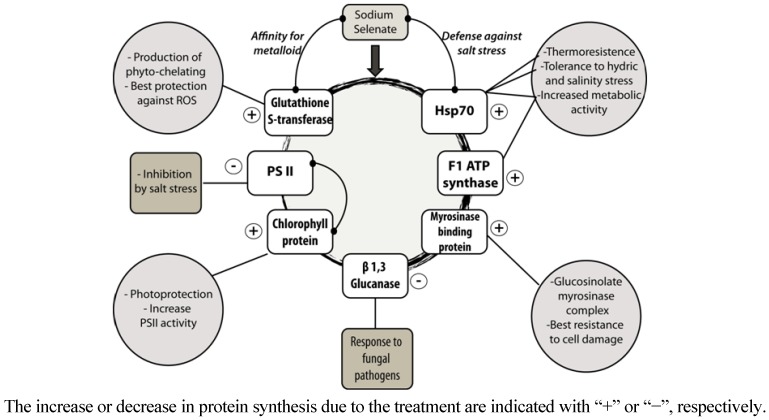
Representation of the main interactions between the proteins that exhibited significant differences in their synthesis with respect to the control condition, due to selenium fortification.

The model depicted in Figure 3 shows the main interactions between the proteins that exhibited significant differences in their synthesis with respect to the control condition, due to selenium fortification. Besides, the model presents the association of those proteins with physiological responses of the plant to different types of stress. In Figure 3, the increase or decrease in protein synthesis due to the treatment are indicated with “+” or “−“, respectively. 

The metabolic pathways where the protein HSP70 participates are related with maintenance of the integrity of protein processing in the plant [20]. An over-synthesis of HSP70 would have a direct effect on the defense capacity of the plant, since it promotes the synthesis of proteins that participate in reparation of oxidative damage produced by Reactive Oxygen Species (ROS). 

The increase of the synthesis of ATP—synthase would be directly related with the over-synthesis of HSP70, since a higher activity of the protein synthesis machinery requires a higher energy input in the cell [21].

β-1,3-Glucanase plays different roles in the plant cells, such as degradation of cell wall (hydrolysis of glucans), however its activity is modulated by the presence of vegetal hormones, pathogenic fungi, and other stress factors [22].

Myrosinase-binding protein, chlorophyll protein and photosystem II are an integral part of the defense system of the plant against stress [23]. Myrosinase-binding protein belongs to the glucosinolate–myrosinase defense complex, which also provides metabolites of high added value that are usually present in *Brassicaceae* [24]. Photosystem II exhibits a negative regulation as stress response, especially stress triggered by photo-oxidation. Chlorophyll protein forms part of the photosynthesis protection system, and it regulates metabolic pathways related with biotic and abiotic stress [25]. As a consequence, an increase in the synthesis of chlorophyll protein would result in a better defense of the plant against stress. 

Glutathione-*S*-transferase plays a crucial role in the defense against stress triggered by heavy metals and metalloids, such as selenium [26]. It is involved in the detoxification system of the plant through the synthesis of chelating complexes that scavenge metals and metalloids, thus avoiding their accumulation and reducing the damage to the plant. Besides, glutathione-*S*-transferase participates in the chemical conversion of metals and metalloids into organic, non-toxic compounds, some of them offering important health benefits to consumers, such as seleno amino acids. 

## 3. Experimental

### 3.1. Broccoli Culture

Heritage broccoli (*Brassica oleracea* var. *italica*) was grown in a greenhouse in individual 9-L pots containing organic soil. Seedlings (10 cm height) were purchased from a local nursery garden. Selenium fortification consisted in adding 30 mL of a 30 mmol/L sodium selenate solution to yield a final concentration of 100 μmol/L sodium selenate in the pot. This procedure began 2 weeks after transplantation and was repeated once a week during 10 weeks. Broccoli florets were harvested when they reached appropriate size, and were kept at −20 °C in plastic sealed bags until analysis. Control plants were included in the study, which received no selenium fortification and were grown in the same conditions that the fortified ones. 

### 3.2. Quantification of SeMSC

The content of Se-methyl selenocysteine (SeMSC) was determined as described by Lyi *et al.* [5]. Broccoli inflorescence (0.1 g of fresh tissue) was pulverized with liquid nitrogen in a mortar, 17 mM HCl (1 mL) was added and amino acids were extracted for 16 h at 4 °C. The mixture was centrifuged at 12,000 × *g* for 10 min and the supernatant was recovered. Amino acids were derivatized with the AccQ-Fluor Reagent Kit (Waters, Milford, MA, USA), separated in a C-18 reverse-phase column (5 μm particle size, 4.6 mm inner diameter, 15 cm length) using an Agilent 1100 series HPLC system (Agilent Technologies, Santa Clara, CA, USA). The fluorescence of derivatized-SeMSC was detected using an emission wavelength of 250 nm and absorption wavelength of 395 nm. SeMSC was identified by co-elution with pure SeMSC (Sigma-Aldrich, Milwaukee, WI, USA) and the concentration was calculated using a calibration curve. 

### 3.3. Quantification of Total Glucosinolates

The content of total glucosinolates was determined as described by Hsu *et al.* [14] with some modifications. Broccoli inflorescence (0.1 g of fresh tissue) was pulverized with liquid nitrogen in a mortar and acidified methanol (280 μL, 40% methanol and 0.5% acetic acid) were added to the ground powder in order to prevent hydrolysis of endogenous glucosinolates by myrosinase (blank sample). The same procedure was performed and water (280 μL) was added to the ground powder to allow the release of glucose from glucosinolates by myrosinase (experimental sample) and the mixture was incubated at 37 °C for 10 min. To stop the reaction, 100% methanol (210 μL) and activated carbon (2 g) were added to precipitate phenolic compounds. The mixture was centrifuged twice at 12,000 × *g* at 4 °C for 10 min and the supernatant was recovered. An aliquot of 100 μL from both supernatants was mixed with 400 μL of glucose assay reagent (Sigma-Aldrich, Milwaukee, WI, USA) and incubated at 37 °C for 30 min and 400 μL of 12-N sulfuric acid were added to stop the reaction. The absorbance of both samples was determined at 540 nm and the absorbance of the blank sample was subtracted to the experimental sample. 

### 3.4. Quantification of Sulforaphane

Sulforaphane content was determined as described by Liang *et al.* [27] with some minor modifications. Broccoli inflorescence (1 g of fresh tissue) was pulverized with liquid nitrogen in a mortar, methylene chloride (10 mL) was added and isothiocyanates were extracted for 30 min at 4 °C. The mixture was supplemented with anhydrous sodium sulfate (2.5 g), filtered through Miracloth paper and dried under vacuum using a rotary evaporator (RE300, Stuart, Staffordshire, UK) at 30 °C. The residue was dissolved in acetonitrile (1 mL) and filtered through a 0.22 μm membrane filter. Isothiocyanates were separated using an Agilent model 1110 HPLC system and a reversed-phase C-18 column (particle size 5 μm, inner diameter 4.6 mm, 15 cm length) at 30 °C. Isothiocyanates were eluted with 20% acetonitrile in water, the solution was then changed linearly over 10 min to 60% acetonitrile and maintained at 100% acetonitrile for 2 min with a flow rate of 1 mL min^−1^. The absorbance of sulforaphane was detected at 254 nm and it was identified by co-elution with pure sulforaphane (Sigma-Aldrich, USA) and the concentration was calculated using a standard curve. 

### 3.5. Protein Extraction and Quantification of Myrosinase Activity

Protein extraction and myrosinase activity was determined as described by Rakariyatham *et al.* [28]. Broccoli inflorescence (0.1 g of fresh tissue) was pulverized with liquid nitrogen in a mortar and buffer 50 mM Tris-HCl pH 5 plus 25 mM EDTA (1 mL) was added. The mixture was centrifuged at 12,000 × *g* at 4 °C and the supernatant was recovered. Protein concentration of the extract was determined using Bradford reagent. Myrosinase activity was determined in reaction mixture (1 mL) containing 1 mM sinigrin, 17 mM sodium sulfate, 0.3 mM magnesium chloride, 0.05 mM ATP, 3.5 U of hexoquinase, 1.75 U of glucose 6-phosphate dehydrogenase, 0.07 mm NADP and 10 μg of protein. The absorbance increase associated to NADPH formation was monitored at 340 nm for 3 min. Myrosinase activity was calculated using the extinction coefficient of NADPH (ε = 6.2 mM^−1^ cm^−1^).

### 3.6. Protein Extraction for 2-D Gel Electrophoresis

Protein extraction from broccoli florets was carried out following the protocol reported by Wang *et al*. [29], with some modifications. To obtain a dry powder, broccoli florets were ground in liquid nitrogen using a mortar and pestle. The powdered tissue was placed in microtubes (0.2 g tissue powder per 1.5 mL microtube) and then resuspended in TCA (1.0 mL). After overnight incubation at 4 °C, tubes were centrifuged at 10,000 × *g* for 20 min, the supernatant was discarded and the pellet was rinsed with cold 10% TCA in acetone five times, then with cold aqueous 10% TCA twice and with cold 80% acetone twice. Protein pellet was air dried at room temperature for 60 min and kept at −20 °C, until extraction. Proteins were extracted using phenol extraction method. To this end, proteins (100 mg) were placed in a 2.0 mL microtube, phenol solution (800 μL, Tris-buffered, pH 8.0; Sigma, St. Louis, MO, USA) and dense SDS buffer (800 μL, 30% sucrose, 2% SDS, 0.1 M Tris-HCl, pH 8.0, 5% 2-mercaptoethanol) were added, tubes were vortexed and then centrifuged. The upper phase (400 μL) was precipitated with cold acetone containing 0.1 M ammonium acetate for 30 min at −20 °C. The pellet was washed three times with 80% acetone, left air dry at room temperature, and stored at −20 °C until analysis. Protein concentration was determined using the Bradford method [30]. 

### 3.7. Two-dimensional Gel Electrophoresis

Two-dimensional (2D) gel electrophoresis was performed as described by Mahn and Ismail [31]. Briefly, protein (300 µg) was resuspended in 50 µl of lysis buffer (9.5 M urea, 2% Triton X-100, 1.6% ampholytes pH range 4–7, 0.4% ampholytes pH range 3–10, and 5% β-mercaptoethanol), incubated at room temperature for 15 min and loaded onto lab-made first-dimension gels (115-mm-height and 3-mm-internal diameter capillary tubes) and a pH gradient of 4.0–7.0 was used. Gel pre-focusing was carried out at 200 V for 15 min, 300 V for 15 min and 400 V for 15 min. Isoelectric focusing was performed at 400 V for 20 h to complete 8,000 Vh. After isoelectric focusing (IEF), the gels were extruded and equilibrated immediately in equilibration solution (2 mL, 10% glycerol; 5% β-mercaptoethanol; 2.3% SDS; 0.0625 M Tris-HCl, pH 6.8) for 10 min. Vertical SDS-PAGE was run with lab-made homogeneous acrylamide gel (11.5% acrylamide; 180 mm in height and 140 mm wide) at a constant voltage of 50 V during 16 h. Gels were fixed in a 25% methanol-7% acetic acid solution for 30 min, stained with Coomassie Brilliant Blue R-250 for 12 h (0.1% Coomassie blue R250, 25% methanol, 7.5% acetic acid) and destained with a 25% methanol and 7.5% acetic acid solution. All chemicals were of analytical grade and were purchased from Sigma (St. Louis, MO, USA). Four independent experiments were carried out for each sample.

### 3.8. Image Acquisition and Analysis

Image acquisition was performed using an ImageScanner II device (GE Healthcare, Uppsala, Sweden). Intensity calibration was carried out with an intensity step wedge prior to gel image capture. Image analysis was carried out using the software Bionumerics (Applied Maths, Inc., Austin, TX, USA). Spots were automatically detected and matched. Only the statistically reproducible spots, *i.e.*, the spots that were present in all gels of the same condition, were considered for further analysis. Each spot volume was processed by background subtraction, and spot volumes of all gels were normalized to remove non-expression-related variations in spot volume. The raw quantity of each spot in a gel was divided by the total quantity of all the valid spots in that gel, as recommended in literature [32,33]. 

### 3.9. Protein Identification

Protein spots were excised directly from the gels and were analyzed by Matrix-Assisted-Laser-Desorption-Ionization/Time-Of-Flight (MALDI-TOF) mass spectroscopy at the Central Proteomics Facility of Sir William Dunn Pathology School at Oxford University (Oxford, UK). Samples were de-stained using a 25 mM ammonium bicarbonate in 50% acetonitrile/water (Mili-Q grade) solution. De-stained gel pieces were reduced with 10 mM DTT and alkylated with 55 mM iodoacetamide. Reduced and alkylated gel pieces were washed 3 times with solution of 25 mM ammonium bicarbonate in 50% acetonitrile/water and dehydrated with 100% acetonitrile before adding 400 ng of Promega Sequencing grade modified trypsin. Samples were left for 1 h at 4 °C to re-hydrate. A 25 mM ammonium bicarbonate solution was added to cover the gel pieces and the samples were incubated at 37 °C over night. Digestion was stopped with 1 μL acetic acid, the supernatant was placed in new low binding microtubes. The gel pieces were covered with 50% acetonitrile/water with 2% formic acid solution and sonicated for 30 min to extract more peptides. The supernatant was added to new microtubes and dried down in a vacuum centrifuge. 

Samples were resuspended in 10 μL 0.1% TFA and desalted using Millipore C18 ZipTips. Samples were eluted in 3 μL of 0.1% TFA in 50% acetonitrile/water solution; 0.5 μL of the eluent was spotted on to Maldi plate and left to air dry. Dried samples were overlaid with 0.5 μL of maldi matrix α-Cyano-4-hydroxycinnamic acid and left to air dry. Then dried samples were analyzed on AB Sciex 4800 Maldi ToF-ToF. The instrument was calibrated using 4700 calibration mixture and after that, samples were analyzed using MS Reflector Positive (for MS spectra) and MS/MS 1kV positive (for MS/MS spectra) methods. The data was searched by GPS explorer software, using Mascot search engine, NCBI Nr Green Plants database.

### 3.10. Statistical Analyses

Statistically significant differences in protein concentration between the selenium-fortified and control broccoli were determined by a Student’s t test at a 95% confidence level. The statistical analyses were performed with Statgraphics^TM^ Plus 5.1 (Statistical Graphics Corp., Princeton, NJ, USA). 

## 4. Conclusions

The SeMSC content in broccoli florets increased significantly with sodium selenate fortification, in comparison with the control plants. Total glucosinolates and sulforaphane content were not affected by selenate fortification, probably due to the comparatively low concentration of sodium selenate in with respect to with the usual sulfur concentration in soil. Myrosinase activity was not affected by selenium fortification, agreeing with the behavior observed for glucosinolates and sulforaphane. The protein profile of broccoli florets was changed due to fortification with sodium selenate-up-regulated proteins were involved in general stress-responses, whereas down-regulated proteins were identified as proteins involved in protection against pathogens. This is the first attempt to evaluate the physiological effect of fortification with sodium selenate on broccoli at protein level. 
